# Self-Medication Practices with Antibiotics among Tertiary Level Students in Accra, Ghana: A Cross-Sectional Study 

**DOI:** 10.3390/ijerph9103519

**Published:** 2012-10-05

**Authors:** Eric S. Donkor, Patience B. Tetteh-Quarcoo, Patrick Nartey, Isaac O. Agyeman

**Affiliations:** 1 Department of Microbiology, University of Ghana Medical School, Accra, Ghana; Email: patborket2002@yahoo.com; 2 Department of Science Laboratory Technology, Accra Polytechnic, P.O. Box GP 561, Accra, Ghana; Email: determino@gmail.com (P.N.); gyenike@yahoo.com (I.S.A.)

**Keywords:** self medication, antibiotics, Ghana

## Abstract

The study was carried out to estimate the prevalence of self-medication with antibiotics among tertiary level students in Accra (Ghana) and evaluate factors associated with the practice. This was a descriptive cross-sectional study and involved face-to-face interviews of 600 respondents selected by convenient sampling. Prevalence of self medication was 70% (95% CI: 66.3–73.7), and the practice was significantly lower among medically inclined students (OR: 0.2, 95% CI: 0.1–0.4, *p* < 0.001). Among the respondents who practiced self medication, the most common frequency of antibiotic usage was at intervals of one month (30%, 95% CI: 25.6–34.4%), and the most common antibiotic used was amoxacillin (23.9%, 95% CI: 21.0–26.8%). Treatment failure were reported by 35% (95% CI: 30.5–39.6%) of the respondents, and the main reasons cited for self medication were that, it was less expensive compared to medical care in the hospital and secondly, medical care in hospitals were associated with long delays. Forty nine percent (95% CI: 44.2–53.8%) of the respondents had poor knowledge about the health implications of irrational use of antibiotics, and 46% (95% CI: 41.2–50.8%) did not comply with the completion of the full course of antibiotics. Self medication among tertiary students in Accra is an important public health problem and this may reflect the situation among tertiary students in the whole of Ghana.

## 1. Introduction

Self-medication can be defined as the use of drugs to treat self-diagnosed disorders or symptoms, or the intermittent or continued use of a prescribed drug for chronic or recurrent disease or symptoms [1]. Self-medication with antibiotics constitute a major form of irrational use of medicine and can cause significant adverse effects such as resistant microorganisms, treatment failures, drug toxicity, increase in treatment cost, prolonged hospitalization periods and increase in morbidity [2,3,4]. Antibiotics represent one of the most prescribed drugs worldwide and their resistance is a major public health threat, hence the need for research on antibiotic usage patterns to help develop appropriate interventions. Studies have shown that self medication with antibiotics is generally prevalent in the developing world [4] and also in some developed countries such as Greece [5]. The prevalent trend of self medication in the developing world has been associated with several factors, particularly, lack of access to health care, availability of antibiotics as over the counter drugs, poor regulatory practices and the relatively higher prevalence of infectious diseases [6,7,8]. 

One of the documented predictors of self medication is level of education [9,10], and several studies have investigated self medication among tertiary level students in different parts of the world [11,12,13,14,15,16,17], though there is no study like that in Ghana. Investigation of self-medication among tertiary level students is important as this population constitute a segment of the society that is highly educated and more inclined to information about health. Of particular importance is the study of self medication among medical or clinical students who represent the future generation of drug prescribers and health educationists.

While irrational use of antibiotics through self medication tends to carry more significance in the developing world, the problem has been investigated in only a few of these countries. In Ghana, a wide range of antibiotics are available on the market and acquiring drugs over the counter is a very common practice [18]. This can facilitate self medication which is thought to be highly prevalent in the Ghanaian community [19], though there are hardly any studies to support this. Recent studies have shown a high level of drug resistance among pathogens to many of the antibiotics available on the Ghanaian market [20,21,22]. This could result in treatment failures and several clinical complications for people practicing self medication. To help address these problems, and also provide a basis for relevant policy measures, the study was undertaken. The objectives of the study were to estimate the prevalence of self-medication with antibiotics among tertiary level students in Accra and evaluate factors associated with self-medication.

## 2. Methods

The study was carried out in Accra, the capital city of Ghana, from September, 2007 to April, 2008. Accra is located in South-Eastern Ghana and has a population of about two million people [23]. The city has about 27 hospitals and most likely over 200 pharmacy shops, each of which is normally manned by a qualified pharmacist [18,24]. With the introduction of national health insurance scheme in Ghana, healthcare is now affordable by most people living in Accra. Nevertheless the chronic problem of high patient to physician ratio can result in severe delays at healthcare centres. Generally, the quality of healthcare provided by private hospitals and pharmacies in Accra is thought to be superior to that provided by the public sector. 

There are seven recognized tertiary institutions in Accra which differ significantly in the study programmes offered. In the current study, a stratified sampling methodology was employed to select four institutions representing the different types of tertiary institutions in Accra. Geographically, the selected institutions adequately cover Accra and included Accra Polytechnic, Central University, Methodist University and Korle-Bu Medical Campus. Based on 95% confidence limits with an allowable error of 10%, numbers of students to be included in the study from each institution were determined as follows: Accra Polytechnic (100), Central University (200), Methodist University (200), Korle-Bu Medical Campus (100). Thus an overall population of 600 students were recruited and this was stratified by gender including the same number (300) of males and females; sample size determination were done at a significance level of 5% and standard power of 0.8 [25,26]. Due to limitations in the set-up of the various institutions, it was not possible to recruit the study subjects by randomization; the subjects were thus selected by convenient sampling involving gender matching. The study was a cross sectional study and the response rate was 90%. No incentives were offered to the study participants.

The questionnaire used in the study comprised 18 closed and open ended questions, and the respondents were first asked if they had ever practiced self medication in their lifetime? Those who replied in the affirmative were further interviewed in two broad areas which included antibiotic usage patterns and health risks of self medication. Under antibiotic usage pattern, the respondents were asked to indicate how often they practiced self medication and which medical conditions they treated. This section also investigated reasons for self medication, which types of antibiotics were used and whether the respondents completed the full course of antibiotics. For the types of antibiotics used in self medication, the respondents were asked to choose from a list of 14 antibiotics and also indicate other antibiotics they used which were not included in the list provided. Under the section on risk of self medication, the respondents were asked to indicate if their antibiotic treatments were successful or not, and in the case of unsuccessful treatment what their next line of action was. This section also investigated whether the respondents were aware of the fact that frequent use or misuse of antibiotics could lead to antibiotic resistance. Prior to commencement of data collection, the study questionnaire was pretested with fifteen respondents to evaluate its validity. This necessitated modification of some of the questions, and the modified version of the questionnaire was also pretested to ensure its validity before actual interviews were done. The questionnaire was administered by two undergraduate students who were trained in the interview process and about how to avoid biases [27]. The questionnaire was in English and on the average it took about 15 minutes to complete.

The data collected were entered into MS Excel and analysed in STATA 7.0 (Strata Corp., College Station. TX, USA). Descriptive analysis was carried out on the study variables and prevalence rates were reported as percentages and 95% confidence intervals. Chi-square was used to evaluate significant association among the study variables and *p* values of <0.05 were considered statistically significant. Specific analyses were carried out to determine:

- prevalence of self medication with antibiotics
- association of self medication with gender and institution
- frequency/rate of self medication
- major antibiotics used in self medication
- causal factors associated with self medication
- prevalence of treatment failures in self medication
- the extent of risk associated with self medication

The protocol of the study was approved by the Institutional Review Board of Accra Polytechnic and informed consent was obtained from study subjects before interviews were done.

## 3. Results

In this study, six hundred tertiary students comprising three hundred each of males and females were sampled from four tertiary institutions and further information on the sampling with regard to the background of the institutions is shown in Table 1. 

**Table 1:** Background information of tertiary institutions where the study sampling was done.

Institution	Type	Major courses offered *	Students sampled
Accra Polytechnic	State owned	Engineering, Sciences, Business	100
Central University	Private owned	Theology, Business, Arts	200
Methodist University	Private owned	Social studies, Arts, Business	200
Korle-Bu Medical Campus	State owned	Medicine, Nursing, Allied Health	100

* represent major courses or programmes of the various institutions at the time of sampling.

Four hundred and twenty two of the respondents (70%) indicated that they practiced self medication with antibiotics. Prevalence of self medication by gender and institution is reported in Table 2, and shows that self medication was significantly associated with institution (*p* < 0.001) but not gender (*p* = 0.08).

**Figure 1:**
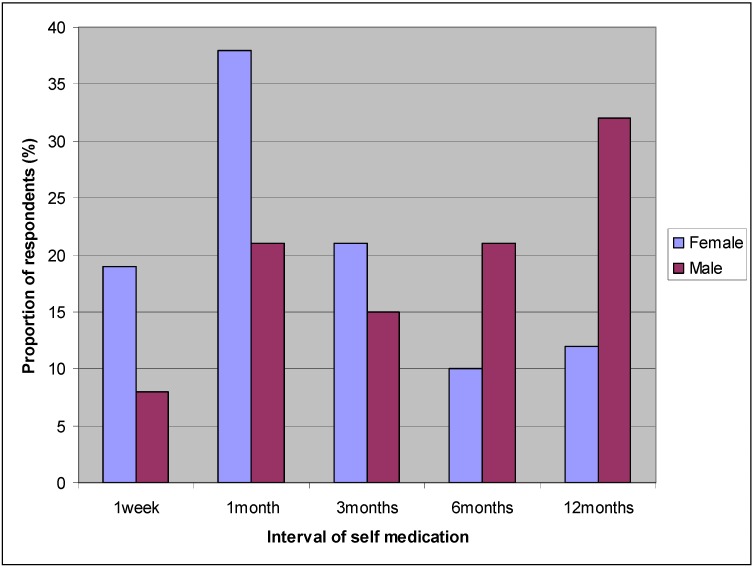
Rate of self medication among male and female respondents.

Analysis of the frequency of self medication (interval of using the same antibiotic) among the respondents showed the following pattern: 14% (59) practiced self medication at intervals of 1 week; 30% (127) practiced self medication at intervals of 1month; 18% (76) practiced self medication at intervals of 3months; 15% (63) practiced self medication at intervals of 6 months; 21% (89) practiced self medication at intervals of 12 months; and 2% (8) practiced self medication at intervals beyond 12 months. The main medical conditions treated in self medication by the respondents were cold, cough, fever and abdominal pains; this was the case even for respondents who practiced self medication at very short intervals (one week). Frequency of self medication was significantly associated with institution (*p* < 0.001) and gender (*p* < 0.001). From Figure 1 it can be seen that self medication at relatively short intervals (weekly, monthly and trimonthly) were more common among females while self medication at relatively long intervals (≥6 months) were more common among males.

**Table 2:** Prevalence of self medication.

Source	n	N	%	95% CI
***Gender* (*p* = 0.08)**				
Male students	202	300	67	61.7–72.3
Female students	220	300	73	68.0–78.0
***Total***	422	600	70	66.3–73.7
***Institution* (*p* < 0.001)**				
Accra Polytechnic	62	100	62	52.5–71.5
Central University	164	200	82	76.7–87.3
Methodist University	154	200	77	71.2–82.8
Korle-Bu Medical Campus	42	100	43	33.3–52.7
***Total***	422	600	70	66.3–73.7

n indicates number of respondents who practice self medication; N indicates total number of respondents; % indicates prevalence of self medication.

Several reasons were cited for practicing self-medication (Table 3). The most important reasons for practicing self medication were that it was less expensive compared to medical care in the hospital (40.5%), and secondly, medical care in hospitals were associated with long delays (40.5%). Inaccessibility of hospitals was the least reason for self medication (0.9%).

Overall, thirteen types of antibiotics were used in self medication by the respondents (Table 3). The most commonly used antibiotic was amoxicillin (23.9%), followed by ampicillin (23%), penicillin (15%) and gentamicin (10.3%). Antibiotics such as vancomycin, sulphonamide and polymyxin were hardly used in self medication (Table 3). 

A proportion of 35% (148) of the students who practiced self-medication indicated that their treatments were not successful, 30% (127) were unsure whether treatments were successful or not, while 35% (148) indicated that treatments were successful. In the case of unsuccessful treatment 23% (97) of the respondents indicated that they revisited the hospital, while the remaining 77% (325) continued with self medication but changed the antibiotic. Fifty one percent of the respondents (215) indicated that they always completed the full course of antibiotics, 46% (194) did not complete the full course of antibiotics while 3% (13) were unsure whether they completed the full course of antibiotics or not. While 51% (215) of the respondents were aware that self medication could cause adverse health effects such as antibiotic resistance, 41% (173) of the respondents had little knowledge about this, while 8% (34) of them were not aware at all of any health risk associated with self medication.

**Table 3:** Antibiotics used and reasons cited for self medication.

Parameter	n	%	95% CI
***Antibiotic type***			
Amoxacillin	198	46.9	42.1–51.7
Ampicillin	191	45.3	40.6–50.1
Penicillin	124	29.4	25.1–33.8
Gentamicin	85	20.1	16.3–23.9
Chloramphenicol	63	14.9	11.5–18.3
Streptomycin	62	14.7	11.3–18.1
Trimethoprim	36	8.5	5.8–11.2
Tetracycline	36	8.5	5.8–11.2
Cotrimoxazole	13	3.1	1.5–4.8
Erythromycin	9	2.1	0.7–3.5
Sulphonamide	5	1.2	0.2–2.3
Polymyxin	4	0.9	0–1.8
Vancomycin	3	0.7	0.1–1.5
***Reasons for self medication***			
Less expensive	171	40.5	35.8–45.2
Long delays at clinics/hospitals	171	40.5	35.8–45.3
Application of previous prescription	46	10.9	7.9–13.9
Good knowledge of antibiotics	34	8.1	5.5–10.7
Antibiotics are easily obtained	30	7.1	4.7–9.6
Imitating others in drug usage	4	0.9	0–1.8
Hospital not accessible	4	0.9	0–1.8

The number of respondents who practiced self medication (422) was used as the denominator in the computation of percentages; sum of percentages exceed 100% as many respondents used more than one type of antibiotics or cited more than one reason for self medication.

## 4. Discussion

To the best of our knowledge, this study represents the first published work on irrational antibiotic use through self medication among humans at a community level in Accra (capital city of Ghana). With a prevalence of 70%, data from this study indicates that self medication with antibiotics is a common practice among tertiary level students in Accra. The high community prevalence of self medication (70%) in this study agrees with a hospital based study in Kumasi (another major city in Ghana), in which the prevalence of self medication with antibiotics was 75% [19]. By comparison, studies on self medication with antibiotics among tertiary level students have reported prevalence rates of 36 and 45% in Turkey [28], 53.5% in Nigeria [10], 76% in Pakistan [13] and 45% in Iran [11]. Some other studies on self medication with antibiotics have rather focused on the general community (rather than students) and reported prevalence rates of 74% in Sudan [9], 78% in Greece [5], 46% in Jordan [29], 3% in Denmark [30], 11% in Spain [31], 19% in Malta [32] and 22% in Lithuania [33]. Evidence from the various studies including ours indicate that self medication appears to be relatively higher in the developing world compared to the developed which is not surprising given the free marketing of antibiotics in the former [8]. 

The significantly lower rate of self medication among medically inclined students compared to students who read non-medical courses, contrasts with a study done in Nigeria which showed that self medication was rather higher among medical students [15]. By comparison some studies done in Pakistan [13] and Iran [11] did not show significant differences in self medication between medical and non-medical students. These observations highlight the geographical and cultural variation in determinants of self medication. The lower prevalence of self medication among medically inclined students in this study is of policy significance and shows that health education may be an important tool in addressing the problem of self medication in Ghana. The importance of such health education is also supported by the fact that, as high as 49% of the respondents have poor knowledge about the possible risks associated with irrational antibiotic usage. It is difficult to explain the differences in frequencies of self medication between male and female students. Basically, it stands to reason that female students encountered diseases more frequently than male students, which may be partly due to the female monthly menstrual cycle during which there are hormonal changes and relatively low immunity. At such times in their cycle, females may feel reluctant to visit the hospital and hence their higher frequency of self medication. This is partly confirmed by a study in Nigeria which showed that 25% of women employed antibiotics to treat menstrual symptoms [10]. Some other factors including age and income are known to influence self medication [9,10], but they were not investigated in this study as the subjects did not have incomes and were of similar ages.

It is worth commenting on some of the antibiotics commonly used in self medication in the light of antibiotic resistance of common bacterial pathogens to these drugs in Ghana. Penicillin, ampicillin, cotrimoxazole, tetracycline and chloramphenicol have been generally reported to have high percentage resistance of 73–82% to several common pathogens in Ghana [22]. Thus the use of these drugs in self medication is of concern and may partly explain the treatment failures associated with self medication in this study. Treatment failures observed may also be due to treatment of non-bacterial infections with antibiotics. For example, cold which was one of the common conditions treated with antibiotics may be caused by bacteria or viruses. Generally, treatment failures in self medication was quite high (35%) and highlights some of the potential health risks and economic losses associated with the practice of self medication.

Self medication among the students appear to be more driven by economic factors meaning that the students were unable to pay for the cost of hospital care and therefore practiced self medication which they considered to be cheaper. This observation agrees with studies done in Sudan [9] and Bogotá [34] and implies that providing affordable medical services may be crucial for dealing with the problem of irrational antibiotic associated with self medication. However the medical services should also be convenient for patients in terms of waiting periods, as delays at hospitals/clinics was another major factor associated with self medication. 

The study showed that about 50% of the study respondents do not complete an antibiotic course which is alarming in the light of behavioural practices that promote antibiotic resistance. Antibiotic resistance has been attributed to misuse and overuse of antibiotics which puts selective pressure on bacterial pathogens leading to the emergence and spread of resistance [8,35]. The consequence of this is the switch from relatively cheap drugs to new drugs, which will be more expensive for developing countries such as Ghana. The rational use of antibiotics to limit the increase in bacterial resistance in the developing world is thus of utmost importance. However, enforcement of antibiotic policies in the developing world also require caution as access to health care services in developing countries is often limited and therefore measures such as strict prescription policy might rather exclude the poor from accessing drugs, resulting in increased morbidity from otherwise treatable infections. In the light of this, it appears that a good strategy in the short term for such developing countries would be to provide appropriate training on how to use antibiotics appropriately and effectively during self medication. In the long term, as health care improves in developing countries like Ghana, it would then be necessary to enforce strict antibiotic policies which have yielded positive results in some countries where they were applied. A good example is in Chile, where to control self-medication, the Chilean Ministry of Health has strictly restricted the purchase of antibiotics without medical prescription since 1999 [36]. This action resulted in a 43% decrease in antibiotic use in the outpatient setting [36], which represents an impressive result. 

The nature of the design of the current study did not permit us to investigate the behaviours of medical officers and pharmacists who are key players in antibiotic usage. According to Macfarlane *et al*. [37] people have great expectation for antibiotics and this may push medical officers to prescribing unnecessarily. As matter of fact, in Ghana and many developing countries, antibiotics may be acquired without any prescriptions [19,38,39]. Since antibiotics are mainly sourced from pharmacy shops in Ghana, pharmacy personnel could play a crucial role in controlling the problem of irrational antibiotic usage. However, unfortunately, in Ghana, pharmacies have become so commerce oriented to the detriment of evidence based practice. In addition to the current study, it may be important to investigate the antibiotic related behaviours of medical officers and pharmacy personnels in Ghana, as such a study would provide additional clues to address irrational antibiotic usage in the country.

## 5. Conclusions

Overall, this study has shown that irrational use of antibiotics through self medication appears to be a common practice among tertiary level students in Accra and we hypothesize that this reflects the trend in the whole of Ghana. In a recent study, we also observed a high rate of misuse of antibiotics in animal husbandry in Ghana [40]. There is also some evidence of antibiotic misuse at the hospital level in Ghana [41]. These observations provide a vivid picture about the abuse of antibiotics in Ghana and explain the escalating trend of antibiotic resistance in the country [10,27,28,29,30]. A national commitment to solving the problem of antibiotic misuse and resistance in Ghana is urgently required. This would require massive health education aimed at behavioural change and strict precautions about irrational use of antibiotics where appropriate [42,43,44,45]. The main limitation of this study is that the data collected were self reported which may introduce some bias in the behaviours of the respondents studied.

## References

[1] Kunin C.M. (1978). Problems of antibiotic usage: Definitions, causes and proposed solutions. Ann. Intern. Med..

[2] Nathwani D., Davey P. (1992). Antibiotic prescribing-Are there lessons for physicians?. Q. Med. J..

[3] Goossens H., Ferech M., Vander Stichele R., Elseviers M. (2005). Outpatient antibiotic use in Europe and association with resistance: A cross-national database study. Lancet.

[4] World Health Organization (2000). Guidelines for the Regulatory Assessment of Medicinal Products for Use in Self-Medication; WHODEDM/QSM/001.

[5] Skliros E., Merkouris P., Papazafiropoulou A., Gikas A., Matzouranis G., Papafragos C., Tsakanikas I., Zarbala I., Vasibosis A., Stamataki P., Sotiropoulos A. (2010). Self-medication with antibiotics in rural population in Greece: A cross-sectional multicenter study. BMC Fam. Pract..

[6] Ebert S.C. (2007). Factors contributing to excessive antimicrobial prescribing. Pharmacotherapy.

[7] Friedman C.R., Whitney C.G. (2008). It’s time for a change in practice: Reducing antibiotic use can alter antibiotic resistance. J. Infect. Dis..

[8] Vila J., Pal T. (2010). Update on antibacterial resistance in low-income countries: Factors favouring the emergence of resistance. Open Infect. Dis. J..

[9] Awad A., Eltayeb I., Matowe L., Thalib L. (2005). Self-medication with antibiotics and antimalarials in the community of Khartoum State, Sudan. J. Pharm. Sci..

[10] Sapkota A.R., Coker M.E., Goldstein R.E.R., Atkinson N.L., Sweet S.J., Sopeju P.O., Ojo M.T., Otivhia E., Ayepola O.O., Olajuyigbe O.O., Shireman L., Pottinger P.S., Ojo K.K. (2010). Self-medication with antibiotics for the treatment of menstrual symptoms in southwest Nigeria: A cross sectional study. BMC Public Health.

[11] Sarahroodi S., Arzi A., Sawalha A.F., Ashtarinezhad A. (2010). Antibiotics self-medication among southern Iranian university students. Int. J. Pharmacol..

[12] Ehigiator O., Azodo C.C., Ehikhamenor E.E. (2010). Self-medication with antibiotics among Nigerian Dental Students. Tanzan. Dent. J..

[13] Syed N.Z., Reema S., Sana W., Akbar Z., Talha V., Mehrine S., Wajeeha Y., Saman S., Sarah S. (2008). Self-medication amongst university students of Karachi: Prevalence, knowledge and attitudes. J. Pak. Med. Assoc..

[14] Fadare J.O., Tamuno I. (2011). Antibiotic self-medication among university medical undergraduates in Northern Nigeria. J. Public Health Epidemiol..

[15] Olayemi O.J., Olayinka B.O., Musa A.I. (2010). Evaluation of antibiotic self-medication pattern amongst undergraduate students of Ahmadu Bello University (Main Campus), Zaria. Res. J. Appl. Sci. Eng. Technol..

[16] Awad A.I., Eltayeb I.B. (2007). Self-medication practices with antibiotics and antimalarials among Sudanese undergraduate university students. Ann. Pharmacother..

[17] Yasmin S.M., Ashraf J., Tahira M., Shahla Z., Adnan S. (2011). Self medication among university students of Karachi. JLUMHS.

[18] Van den Boom G.J.M., Nsowah-Nuamah N.N.N., Overbosch G.B. Healthcare Provision and Self-Medication in Ghana. http://web.archive.org/web/20070625163825/http://www.saga.cornell.edu/images/vandenboom.pdf.

[19] Adu-Sarkodie Y.A. (1997). Antimicrobial self medication in patients attending a sexually transmitted diseases clinic. Int. J. STD AIDS.

[20] Donkor E.S., Nartey E. (2008). Nasal colonisation of drug resistant bacteria in Ghanaian children less than five years. Internet J. Microbiol..

[21] Edoh D., Alomatu B. (2008). Comparison of antibiotic resistance patterns between laboratories in Accra east Ghana. Niger. Ann. Nat. Sci..

[22] Newman M.J., Frimpong E., Donkor E.S., Opintan J.A., Asamoah-Adu A. (2011). Resistance to antimicrobial drugs in Ghana. Infect. Drug Resist..

[23] Ghana Statistical Service (2010). 2010 Population and Housing Census. Provisional Results. http://www.ghana.gov.gh/census/phc2010.pdf.

[24] Owusu-Daak F.T., Marfo A.F.A., Boateng E.A. (2010). The contribution of Ghanaian pharmacists to mental health care: Current practice and barriers. Int. J. Ment. Health Syst..

[25] Daniel W.W. (1998). Biostatistics: A Foundation for Analysis in the Health Sciences.

[26] Naing L., Winn T., Rusli B.N. (2006). Practical issues in calculating the sample size for prevalence studies. Arch. Orofac. Sci..

[27] Pannucci C.J., Wilkins E.G. (2010). Identifying and avoiding bias in research. Plast Reconstr. Surg..

[28] Buke C., Hosgor-Limoncu M., Ermertcan S., Ciceklioglu M., Tuncel M., Kose T., Eren S. (2005). Irrational use of antibiotics among university students. J. Infect..

[29] Al-Bakri A.G., Bustanji Y., Yousef A.M. (2005). Community consumption of antibacterial drugs within the Jordanian population: Sources, patterns and appropriateness. Int. J. Antimicrob. Agents.

[30] Muscat M., Monnet D.L., Klemmensen T., Grigoryan L., Jensen M.H., Andersen M., Haaijer-Ruskamp F.M. (2006). Patterns of antibiotic use in the community in Denmark. Scand. J. Infect. Dis..

[31] Väänänen M.H., Pietilä K., Airaksinen M. (2006). Self-medication with antibiotics-Does it really happen in Europe?. Health Policy.

[32] Borg M.A., Scicluna E.A. (2002). Over-the-counter acquisition of antimicrobial drugs in the Maltese general population. Int. J. Antimicrob. Agents.

[33] Berzanskyte A., Valinteliene R., Haaijer-Ruskamp F.M., Gurevicius R., Grigoryan L. (2006). Self-medication with antibiotics in Lithuania. Int. J. Occup. Med. Environ. Health.

[34] López J.J., Dennis R., Moscoso S.M. (2009). A study of self-medication in a neighborhood in Bogotá. Rev. Salud. Publica (Bogota).

[35] Harbarth S., Samore M.H. (2005). Antimicrobial resistance determinants and future control. Emerg. Infect. Dis..

[36] Bavestrello L., Cabello A., Casanova D. (2002). Impact of regulatory measures in the trends of community consumption of antibiotics in Chile. Rev. Med. Chil..

[37] Macfarlane J., Holmes W., Macfarlane R., Britten N. (1997). Influence of patients’ expectations on antibiotic management of acute lower respiratory tract illness in general practice: Questionnaire study. BMJ.

[38] Reeves D. (2007). The 2005 Garrod lecture: The changing access of patients to antibiotics-For better or worse?. J. Antimicrob. Chemother..

[39] Lee P.R., Lurie P., Silverman M.M., Lydecker M. (1991). Drug labelling and promotion in the developing countries: An update. J. Clin. Epidemiol..

[40] Donkor E.S., Newman M.J., Yeboah-Manu D. (2012). Epidemiological aspects of non-human antibiotic usage and resistance: Implications for the control of antibiotic resistance in Ghana. Trop. Med. Int. Health.

[41] Bonsu W.K., Ofori-Adjei D. (2000). An audit of prescribing practice in health care facilities of the Wassa west district of Ghana. West Afr. J. Med..

[42] Donkor E.S., Nortey T., Opintan J.A., Dayie N., Akyeh M.L. (2008). Antimicrobial susceptibility of Salmonella typhi and Staphylococcus aureus and the effect of some media on susceptibility testing results. Internet J. Microbiol..

[43] Donkor E.S., Newman M.J., Oliver-Commey J., Bannerman E., Dayie N.T.K.D., Badoe E.V. (2010). Invasive disease and paediatric carriage of Streptococcus pneumoniae in Ghana. Scand. J. Infect. Dis..

[44] Opintan J.A., Newman M.J. (2007). Distribution of serogroups and serotypes of multiple drug resistant Shigella isolates. Ghana Med. J..

[45] Enweronu-Laryea C.C., Newman M.J. (2007). Changing pattern of bacterial isolates and antimicrobial susceptibility in neonatal infections in Korle Bu Teaching Hospital, Ghana. East Afr. Med. J..

